# Clinical Characteristics and Outcomes of Childbearing-Age Women With COVID-19 in Wuhan: Retrospective, Single-Center Study

**DOI:** 10.2196/19642

**Published:** 2020-08-24

**Authors:** Lijie Wei, Xuan Gao, Suhua Chen, Wanjiang Zeng, Jianli Wu, Xingguang Lin, Huiting Zhang, Lali Mwamaka Sharifu, Ling Chen, Ling Feng, Shaoshuai Wang

**Affiliations:** 1 Department of Obstetrics and Gynecology Tongji Hospital Tongji Medical College, Huazhong University of Science and Technology Wuhan, Hubei 430030 China; 2 Department of Pediatrics Tongji Hospital Tongji Medical College, Huazhong University of Science and Technology Wuhan China

**Keywords:** COVID-19, SARS-CoV-2, childbearing age, pregnancy, clinical characteristics, outcomes, women, health information, epidemiology, diagnosis, symptom

## Abstract

**Background:**

Since December 2019, an outbreak of the coronavirus disease (COVID-19) caused by severe acute respiratory syndrome coronavirus 2 (SARS-CoV-2) has spread rapidly in Wuhan and worldwide. However, previous studies on pregnant patients were limited.

**Objective:**

The aim of this study is to evaluate the clinical characteristics and outcomes of pregnant and nonpregnant women with COVID-19.

**Methods:**

This study retrospectively collected epidemiological, clinical, laboratory, imaging, management, and outcome data of 43 childbearing-age women patients (including 17 pregnant and 26 nonpregnant patients) who presented with laboratory-confirmed COVID-19 in Tongji Hospital, Wuhan, China from January 19 to March 2, 2020. Clinical outcomes were followed up to March 28, 2020.

**Results:**

Of the 43 childbearing-age women in this study, none developed a severe adverse illness or died. The median ages of pregnant and nonpregnant women were 33.0 and 33.5 years, respectively. Pregnant women had a markedly higher proportion of history exposure to hospitals within 2 weeks before onset compared to nonpregnant women (9/17, 53% vs 5/26, 19%, *P*=.02) and a lower proportion of other family members affected (4/17, 24% vs 19/26, 73%, *P*=.004). Fever (8/17, 47% vs 18/26, 69%) and cough (9/17, 53% vs 12/26, 46%) were common onsets of symptoms for the two groups. Abdominal pain (n=4, 24%), vaginal bleeding (n=1, 6%), reduced fetal movement (n=1, 6%), and increased fetal movement (n=2, 13%) were observed at onset in the 17 pregnant patients. Higher neutrophil and lower lymphocyte percent were observed in the pregnant group compared to the nonpregnant group (79% vs 56%, *P*<.001; 15% vs 33%, *P*<.001, respectively). In both groups, we observed an elevated concentration of high-sensitivity C-reactive protein, erythrocyte sedimentation rate, aminotransferase, and lactate dehydrogenase. Concentrations of alkaline phosphatase and D-dimer in the pregnant group were significantly higher than those of the nonpregnant group (119.0 vs 48.0 U/L, *P*<.001; 2.1 vs 0.3μg/mL, *P*<.001, respectively). Both pregnant (4/10, 40%) and nonpregnant (8/15, 53%) women tested positive for influenza A virus. A majority of pregnant and nonpregnant groups received antiviral (13/17, 76% vs 25/26, 96%) and antibiotic (13/17, 76% vs 23/26, 88%) therapy. Additionally, both pregnant (2/11, 18%) and nonpregnant (2/19, 11%) recovered women redetected positive for SARS-CoV-2 after discharge.

**Conclusions:**

The epidemiology and clinical and laboratory features of pregnant women with COVID-19 were diverse and atypical, which increased the difficulty of diagnosis. Most pregnant women with COVID-19 were mild and moderate, and rarely developed severe pneumonia or severe adverse outcomes.

## Introduction

In December 2019, a cluster of cases of pneumonia of unknown etiology were identified in Wuhan, China [1]. Further investigation revealed these cases were caused by a novel coronavirus, which was termed severe acute respiratory syndrome coronavirus 2 (SARS-CoV-2). Pneumonia caused by SARS-CoV-2 was termed the coronavirus disease (COVID-19) [2,3]. In the past 2 decades, two human coronaviruses including severe acute respiratory syndrome–related coronavirus (SARS-CoV) and Middle East respiratory syndrome–related coronavirus (MERS-CoV) can cause severe lower respiratory tract infections [4,5]. SARS-CoV-2 is similar to SARS-CoV as both of them belong to the beta coronavirus genus, and SARS-CoV-2 shares more than 79.6% sequence identity with SARS-CoV [6]. As of April 18, 2020, the cumulative number of confirmed cases of COVID-19 infection in China had exceeded 86,700, and the death toll is more than 4600. The cumulative total number of confirmed cases has globally exceeded 2,350,000 and continues to increase [7,8]. The World Health Organization (WHO) has designated the COVID-19 pandemic a Public Health Emergency of International Concern.

Pregnant women have been hypothesized to be susceptible to respiratory pathogens and severe adverse outcomes of pneumonia due to the normal physiological changes during pregnancy, including altered cell-mediated immunity and changes in pulmonary [9,10]. Previous studies reported that pregnant women infected with SARS-CoV or MERS-CoV were more susceptible to severe adverse outcomes including maternal morbidity and death. The case fatality rate (CFR) for pregnant women infected with SARS-CoV reached 25%-30%, much higher than that of the general population [11,12]. Data for pregnant women infected with MERS-CoV is scarce. A case series of 5 pregnant women with MERS reported that the CFR reached 40% [13]. Unfortunately, there is limited experience on COVID-19 infections during pregnancy, and all current studies are single-center trials. Two studies with a small sample size reported none of the pregnant women with COVID-19 have died yet [14,15]. However, currently there is no vaccine or specific treatment for COVID-19 infection.

In this study, we describe the clinical, laboratory, imaging findings, and clinical outcomes of 43 childbearing-age women patients (including 17 pregnant and 26 nonpregnant women) in Wuhan infected with SARS-CoV-2. This will provide an insight into the prevention and treatment of pregnant women with COVID-19.

## Methods

### Recruitment

This study retrospectively recruited patients from January 19 to March 2, 2020, at Tongji Hospital, Tongji Medical College of Huazhong University of Science and Technology, Wuhan, Hubei, China. According to the arrangements put in place by the Chinese Government, pregnant and nonpregnant women patients were admitted to the designated hospitals in Wuhan for managing COVID-19 without selectivity. All patients were diagnosed with COVID-19 according to “Diagnosis and Treatment Protocol for COVID-19 (Sixth Trial Edition)” released by the National Health Commission of the People’s Republic of China [16].

There were 17 pregnant and 32 nonpregnant women’s throat swabs that tested positive for SARS-CoV-2 RNA from January 19 to March 2, 2020; among them, 6 nonpregnant women with comorbidities were excluded (2 had hypertension, 1 had diabetes, 1 had a history of kidney transplantation, 1 had lymphoma, and 1 had connective tissue disease). The remaining 17 pregnant women and 26 nonpregnant women did not have any underlying comorbidities due to a chronic disease such as hypertension, diabetes, or heart disease. Two groups were matched with respect to age, gender, timing of contact with COVID-19, and the proportion of health care workers. Additionally, all patients recruited were Chinese residents and lived in Wuhan with no exposure to the Huanan seafood market in Wuhan.

This study was reviewed and approved by the Ethics Committee of Tongji Hospital, Tongji Medical College of Huazhong University of Science and Technology (TJ-IRB20200222). Informed consent for this retrospective study was waived. The anonymous data was collected and analyzed to facilitate better clinical decisions and treatment.

### Data Collection

We retrospectively collected epidemiological, clinical, laboratory, imaging, management, and outcome data for all the patients with COVID-19 in the two groups. Clinical outcomes were followed up to March 28, 2020. Two researchers (LW and XG) evaluated the participants and reviewed the data independently, with disagreements resolved by consensus.

Throat swab specimens for all patients were tested for SARS-CoV-2 at Tongji Hospital. SARS-CoV-2 was confirmed following the WHO guidelines for quantitative real-time reverse transcription polymerase chain reaction (qRT-PCR) [17]. Throat swab specimens from the upper respiratory tract that were obtained from all patients on admission were maintained in a viral-transport medium. Other pneumonia-related respiratory pathogens including influenza A virus, influenza B virus, respiratory syncytial virus, adenovirus, parainfluenza viruses, legionella pneumophila, mycoplasma pneumoniae, and chlamydia pneumoniae were tested by enzyme-linked immunosorbent assay (ELISA). qRT-PCR and ELISA detection reagents were provided by Tongji Hospital. Additionally, except for 1 pregnant woman who did not consent, all the remaining patients took a chest computed tomography (CT).

### Statistical Analysis

Statistical analysis was performed with SPSS version 23.0 (IBM Corp). Continuous variables were presented as median (interquartile range). Categorical variables were expressed as number and proportion (%). The Mann-Whitney U test was applied for comparing two groups of continuous variables. The chi-square test or Fisher exact test were applied for discrete variables of two groups. A *P* value with a two-tailed test <.05 was considered as statistically significant.

## Results

### Demographics and Clinical Characteristics of Pregnant Women and Nonpregnant Women

A total of 17 pregnant and 26 nonpregnant women with COVID-19 were included in this study. Among the 17 pregnant women, 1 was in her first trimester, 3 were in their second trimester, and 13 were in their third trimester. None of them had a history of exposure to the Huanan seafood market. Of the sample, 18% (3/17) of pregnant and 19% (5/26) of nonpregnant women were health care workers. Pregnant women had a higher proportion of history exposure to hospitals within 2 weeks before onset compared to nonpregnant women (9/17, 53% vs 5/26, 19%, *P*=.02) and a lower proportion of other family members infected with COVID-19 (4/17, 24% vs 19/26, 73%, *P*=.004) than nonpregnant women. The median ages of pregnant and nonpregnant women were 33.0 and 33.5 years, respectively. The median time from symptom onset to hospital presentation in the pregnant and nonpregnant groups were 2.0 and 4.0 days, respectively. Of the patients, 2 of 17 (12%) pregnant women and 3 of 26 (11%) nonpregnant women were diagnosed with a severe type on admission. None of the patients developed critical illness (Table 1).

The symptoms at onset of pregnant women with COVID-19 were similar to nonpregnant women. The most common symptoms at onset of pregnant and nonpregnant women were fever (8/17, 47% vs 18/26, 69%) and cough (9/17, 53% vs 12/26, 46%). Other pneumonia-related symptoms at onset including fatigue, expectoration, chest tightness, and shortness of breath were less common. Chills and rigors, headache, and myalgia had not been observed in pregnant women prior to the infection. Both pregnant (1/17, 6%) and nonpregnant (4/26, 15%) groups had diarrhea at onset. Of the 17 pregnant women, 2 (12%) asymptomatic pregnant women were diagnosed during hospitalization routine tests as a requirement before delivery. Of the 26 nonpregnant women, 2 (8%) asymptomatic nonpregnant women were diagnosed by testing for SARS-CoV-2 of throat swabs because they had a history of contact with an infected person. Additionally, pregnancy-related symptoms were also observed in pregnant women, including abdominal pain (n=4, 24%), vaginal bleeding (n=1, 6%), reduced fetal movement (n=1, 6%), and increased fetal movement (n=2, 13%). There were 2 pregnant women who only had pregnancy-related symptoms until being diagnosed (Table 1).

**Table 1:** Epidemiological and clinical features of pregnant and nonpregnant women with the coronavirus disease.

Variables		Total (N=43)		Pregnancy (n=17)		Nonpregnancy (n=26)		*P* value	
Age (years), median (IQR)		33.0 (30.0-37.0)		33.0 (30.0-35.0)		33.5 (31.0-38.0)		.28	
**Gestational age on admission, n (%)**									
	First trimester		N/A^a^		1 (6)		N/A		N/A
	Second trimester		N/A		3 (18)		N/A		N/A
	Third trimester		N/A		13 (76)		N/A		N/A
Health care workers, n (%)		8 (19)		3 (18)		5 (19)		.77	
Hospital exposure within 2 weeks before onset, n (%)		14 (33)		9 (53)		5 (19)		.02	
Other family members affected, n (%)		23 (53)		4 (24)		19 (73)		.004	
Time from onset of symptom to first outpatient visit (days), median (IQR)		3.5 (1.0-7.0)		2.0 (0.9-10.8)		4.0 (1.0-7.0)		.75	
**Clinical classification, n (%)**									.54
	Mild		3 (7)		2 (12)		1 (4)		
	Moderate		35 (81)		13 (76)		22 (85)		
	Severe		5 (12)		2 (12)		3 (12)		
	Critical		0 (0)		0 (0)		0 (0)		
**Symptoms at onset, n (%)**									
	Fever		26 (60)		8 (47)		18 (69)		.15
	Chills and rigors		2 (5)		0 (0)		2 (8)		.67
	Headache		1 (2)		0 (0)		1 (4)		.83
	Dizziness		1 (2)		1 (6)		0 (0)		.83
	Fatigue		5 (12)		1 (6)		4 (15)		.93
	Cough		21 (49)		9 (53)		12 (46)		.66
	Expectoration		9 (21)		3 (18)		6 (23)		.96
	Chest tightness		5 (12)		2 (12)		3 (12)		.64
	Shortness of breath		2 (5)		1 (6)		1 (4)		.67
	Myalgia		1 (2)		0 (0)		1 (4)		.83
	Diarrhea		5 (12)		1 (6)		4 (15)		.64
	Asymptomatic		4 (9)		2 (12)		2 (8)		.93
	Abdominal pain		N/A		4 (24)		N/A		N/A
	Vaginal bleeding		N/A		1 (6)		N/A		N/A
	Reduced fetal movements		N/A		1 (6)		N/A		N/A
	Increased fetal movement		N/A		2 (13)		N/A		N/A

^a^N/A: not applicable.

### Laboratory and Imaging Characteristics of Pregnant Women and Nonpregnant Women

On admission, the median white blood cell count of patients in the pregnant group with COVID-19 was significantly higher than the nonpregnant group (7.8 vs 3.8×10^9^/L, *P*<.001). There were 4/17 (24%) pregnant women and 0/26 (0%) nonpregnant women that developed leukocytosis (white blood cell count>10.0×10^9^/L). Neutrophil percentage and neutrophil count were higher in pregnant women compared to nonpregnant women (79% vs 56%, *P*<.001; 6.7 vs 2.3×10^9^/L, *P*<.001, respectively). Lymphopenia (lymphocyte count<1.0×10^9^/L) occurred in 7 (41%) of the 17 pregnant women and 10 (38%) of the 26 nonpregnant women. There was no statistical difference in hemoglobin concentration and platelet count between the two groups (Table 2).

**Table 2:** Laboratory and imaging features of pregnant and nonpregnant women with the coronavirus disease.

Variables				Total (N=43)		Pregnancy (n=17)		Nonpregnancy (n=26)		*P* value	
**Routine blood test**											
	**White blood cell count (×10^9^/L), median (IQR)**		5.2 (3.8-7.6)		7.8 (6.6-10.2)		3.8 (3.6-5.1)		<.001		
		<4.0, n (%)	12 (28)		0 (0)		12 (46)		.003		
		>10.0, n (%)	4 (9)		4 (24)		0 (0)		.04		
	**Neutrophil percent (%), median (IQR)**		64.4 (55.9-79.4)		80.5 (72.2-85.2)		58.0 (49.4-63.0)		<.001		
		>75, n (%)	12 (28)		12 (71)		0 (0)		<.001		
	**Neutrophil count (×10^9^/L), median (IQR)**		3.3 (2.1-5.5)		6.7 (5.3-8.2)		2.3 (1.9-2.9)		<.001		
		<1.5, n (%)	1 (2)		0 (0)		1 (3)		.83		
	**Lymphocyte percent (%), median (IQR)**		24.9(14.4-35.9)		13.0 (11.6-20.1)		32.7 (26.4-39.7)		<.001		
		<20, n (%)	14 (33)		13 (77)		1 (4)		<.001		
	**Lymphocyte count (×10^9^/L), median (IQR)**		1.4 (1.0-1.8)		1.1 (0.9-1.6)		1.4 (1.0-2.0)		.21		
		<1.0, n (%)	17 (40)		7 (42)		10 (38)		.86		
	**Hemoglobin (g/L), median (IQR)**		122.5 (113.8-128.5)		117.0 (111.0-132.0)		123.0 (117.0-127.0)		.86		
		<115, n (%)	12 (28)		6 (35)		6 (23)		.38		
	**Platelet count (×10^9^/L), median (IQR)**		209.0 (160.0-242.0)		198.0 (138.0-227.3)		210.0 (171.0-250.3)		.24		
		<150, n (%)	10 (23)		6 (35)		4 (15)		.25		
**Other laboratory features**											
	**High sensitivity C-reactive protein (mg/L), median (IQR)**		6.7 (0.7-25.3)		16.7 (7.1-47.6)		1.6 (0.4-13.0)		.07		
		≥10, n/N (%)	14/31 (45)		7 /10 (70)		7/21 (33)		.12		
	**Procalcitonin (ng/mL), median (IQR)**		0.04 (0.03-0.05)		0.05 (0.03-0.17)		0.04 (0.03-0.05)		.16		
		≥0.05, n/N (%)	0/23 (0)		0/9 (0)		0/14 (0)		N/A^a^		
	**Erythrocyte sedimentation rate (mm/h), median (IQR)**		26.0 (12.0-41.0)		36.5 (26.3-82.0)		24.0 (7.0-38.0)		.08		
		>20, n/N (%)	13/20 (65)		5/5 (100)		8/15 (53)		.11		
	**Alanine aminotransferase (U/L), median (IQR)**		16.5 (9.0-26.0)		13.0 (9.0-28.0)		23.0 (9.0-26.5)		.72		
		≥45, n/N (%)	6/42 (14)		3/17 (18)		3/25 (12)		.95		
	**Aspartate aminotransferase (U/L), median (IQR)**		17.0 (13.0-28.3)		20.0 (14.0-42.5)		15.0 (10.5-25.0)		.047		
		≥35, n/N (%)	9/42 (21)		5/17 (29)		4/25 (16)		.51		
	**Lactate dehydrogenase (U/L), median (IQR)**		204.0 (172.0-286.0)		235.0 (182.0-309.0)		193.0 (161.0-277.0)		.13		
		≥250, n/N (%)	13/38 (34)		6/15 (40)		7/23 (30)		.73		
	**Alkaline phosphatase (U/L), median (IQR)**		57.5 (46.5-111.3)		119.0 (77.0-142.0)		48.0 (42.0-57.0)		<.001		
		≥100, n/N (%)	11/38 (29)		10/15 (67)		1/23 (4)		<.001		
	**Creatinine (μmol/L), median (IQR)**		52.5 (46.0-61.0)		50.0 (43.2-59.5)		53.0 (48.0-62.5)		.21		
		≥106, n/N (%)	0/38 (0)		0/14 (0)		0/24 (0)		N/A		
	**Creatine kinase (U/L), median (IQR)**		51.5 (35.8-70.8)		81.0 (29.0-147.5)		48.5 (37.3-61.0)		.34		
		≥140, n/N (%)	1/20 (5)		1/6 (17)		0/14 (0)		.30		
	**D-dimer (μg/mL), median (IQR)**		0.7 (0.3-2.0)		2.1 (1.7-3.1)		0.3 (0.2-0.7)		<.001		
		≥0.5, n/N (%)	19/34 (56)		11/12 (92)		8/22 (36)		.003		
**Pneumonia-associated pathogens, n/N (%)**											
	Respiratory syncytial virus		0/24 (0)		0/10 (0)		0/14 (0)		N/A		
	Adenovirus		0/24 (0)		0/10 (0)		0/14 (0)		N/A		
	Influenza A virus		12/25 (48)		4/10 (40)		8/15 (53)		.69		
	Influenza B virus		0/25 (0)		0/10 (0)		0/15 (0)		N/A		
	Parainfluenza viruses		0/24 (0)		0/10 (0)		0/14 (0)		N/A		
	Legionella pneumophila		1/24 (4)		1/10 (10)		0/14 (0)		.42		
	Mycoplasma pneumoniae		2/22 (9)		1/10 (10)		1/12 (8)		>.99		
	Chlamydia pneumoniae		0/23 (0)		0/10 (0)		0/13 (0)		N/A		
**Chest computed tomographic findings, n/N (%)**											.67
	Normal		1/42 (2)		0/16 (0)		1/26 (4)				
	Unilateral pneumonia		9/42 (21)		3/16 (19)		6/26 (23)				
	Bilateral pneumonia		32/42 (76)		13/16 (81)		19/26 (73)				

^a^N/A: not applicable.

In both pregnant and nonpregnant groups, we observed elevated high-sensitivity C-reactive protein (hs-CRP; ≥10 mg/L: 7/10, 70% vs 7/21, 33%) and erythrocyte sedimentation rate (>20 mm/hr: 5/5 100% vs 8/15, 53%). The mean concentrations of alanine aminotransferase (ALT) or aspartate aminotransferase (AST) in the pregnant group were above the normal range, while the nonpregnant group was normal. One patient in the pregnant group had ALT of up to 882 U/L and AST of up to 783 U/L. Concentration of lactate dehydrogenase (LDH) and alkaline phosphatase in the pregnant group were observed as higher than in the nonpregnant group (235.0 vs 193.0 U/L; 119.0 vs 48.0 U/L, respectively). Additionally, 92% (11/12) of pregnant women were observed with an elevated D-dimer level, which was significantly higher than nonpregnant women (2.1 vs 0.3 μg/mL, *P*<.001; Table 2).

**Figure 1:**
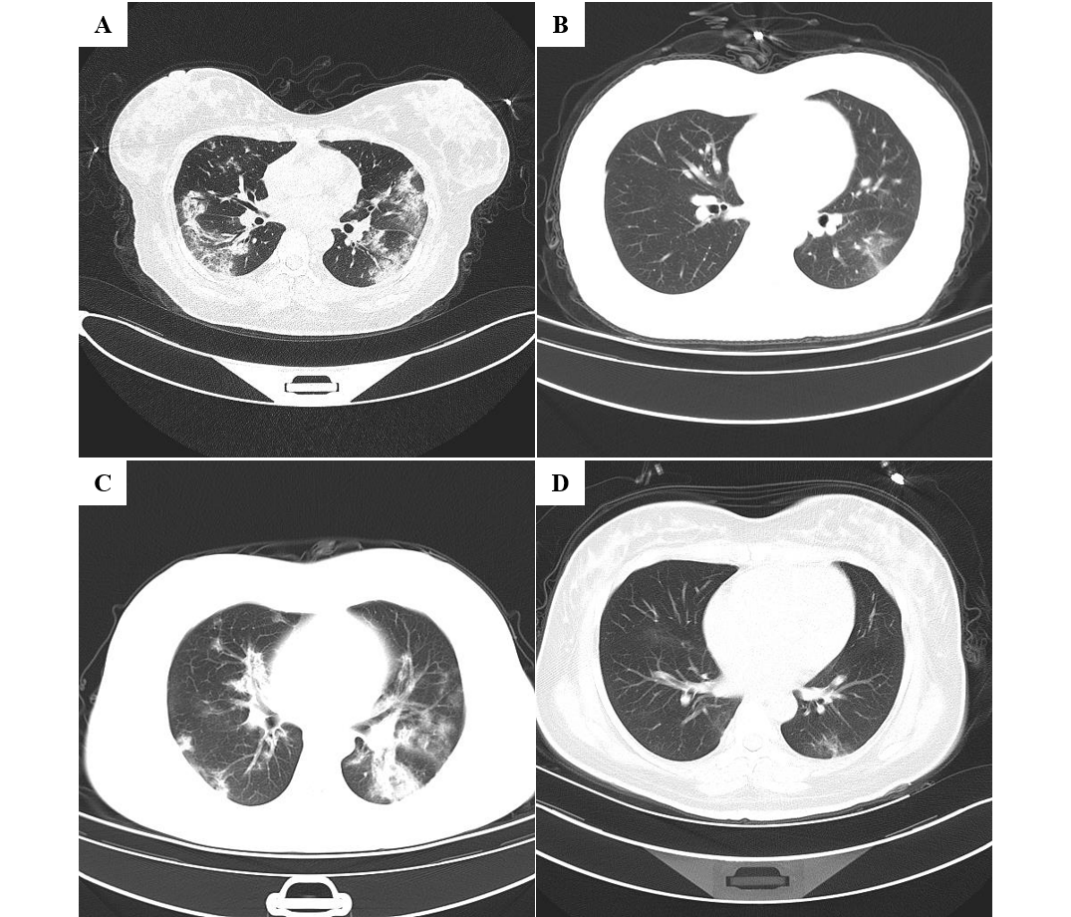
Chest computed tomography scans of 4 patients with the coronavirus disease.

Serological examination of pneumonia-associated pathogens was performed in pregnant and nonpregnant patients with COVID-19. There were 4/10 (40%) pregnant women and 8/15 (53%) nonpregnant women that tested positive for influenza A virus immunoglobulin M. Other respiratory viruses had not been observed. Of 10 pregnant women, 1 (10%) tested positive for legionella pneumophila and 1 (10%) tested positive for mycoplasma pneumoniae. Except for 1 pregnant woman who refused to undergo a chest CT scan, all patients accepted chest CT examinations. Of the 42 patients, 41 (98%) displayed typical findings of pneumonia, in which 9 (21%) patients had unilateral pneumonia and 32 (76%) patients had bilateral pneumonia (Table 2 and Figure 1).

In Figure 1, A and B are chest CTs showing the axial view lung window of 2 pregnant women with COVID-19. A is the chest CT from a 34-year-old woman who was 38 weeks and 4 days pregnant, showing multiple bilateral ground-glass opacities. B is a chest CT from a 30-year-old woman who was 39 weeks and 1 day pregnant, showing left-sided ground-glass opacity. C and D are chest CTs showing the axial view lung window of 2 nonpregnant women with COVID-19. C is a chest CT from a 30-year-old woman showing multiple bilateral ground-glass opacities. D is a chest CT from a 33-year-old woman showing left-sided ground-glass opacity.

### Management and Clinical Outcomes of Pregnant Women and Nonpregnant Women

A majority of the 17 pregnant and 26 nonpregnant patients with COVID-19 received antiviral (n=13, 76% vs n=25, 96%) and antibiotic (n=13, 76% vs n=23, 88%) therapy. There were 4 (24%) pregnant and 5 (19%) nonpregnant women that received glucocorticoid therapy, and 1 (6%) of the pregnant and 3 (12%) of the nonpregnant women received immunoglobulins therapy. Compared with the pregnant group, the proportion of patients that received antitussive therapy in the nonpregnant group significantly increased (18/26, 69% vs 6/17, 35%, *P*=.03). Additionally, oxygen support was administered in 35% (6/17) of pregnant and 54% (14/26) of the nonpregnant women with COVID-19. None of the patients underwent mechanical ventilation, continuous renal replacement therapy, or extracorporeal membrane oxygenation (Table 3).

None of the patients were lost in the follow-up during the study. None of the patients in the two groups were admitted to the intensive care unit (ICU), and none developed acute respiratory distress syndrome, disseminated intravascular coagulation (DIC), renal failure, heart failure, secondary bacterial pneumonia, or sepsis. In addition, none of the patients died (Table 3).

**Table 3:** Clinical treatment and outcomes of pregnant and nonpregnant women with the coronavirus disease.

Variables			Total (N=43)	Pregnancy (n=17)	Nonpregnancy (n=26)	*P* value
**Management, n (%)**						
	Antiviral therapy		38 (88)	13 (76)	25 (96)	.14
	Antibiotic therapy		36 (84)	13 (76)	23 (88)	.54
	Glucocorticoid therapy		9 (21)	4 (24)	5 (19)	.96
	Immunoglobulin		4 (9)	1 (6)	3 (12)	.93
	Cough-suppressant therapy		24 (56)	6 (35)	18 (70)	.03
	Oxygen support (nasal cannula)		20 (47)	6 (35)	14 (54)	.23
	**Mechanical ventilation**		0 (0)	0 (0)	0 (0)	N/A^a^
		Noninvasive	0 (0)	0 (0)	0 (0)	N/A
		Invasive	0 (0)	0 (0)	0 (0)	N/A
	Continuous renal replacement therapy		0 (0)	0 (0)	0 (0)	N/A
	Extracorporeal membrane oxygenation		0 (0)	0 (0)	0 (0)	N/A
**Clinical outcomes**						
	Intensive care unit admission, n (%)		0 (0)	0 (0)	0 (0)	N/A
	Acute respiratory distress syndrome, n (%)		0 (0)	0 (0)	0 (0)	N/A
	Disseminated intravascular coagulation, n (%)		0 (0)	0 (0)	0 (0)	N/A
	Renal failure, n (%)		0 (0)	0 (0)	0 (0)	N/A
	Heart failure, n (%)		0 (0)	0 (0)	0 (0)	N/A
	Secondary bacterial pneumonia, n (%)		0 (0)	0 (0)	0 (0)	N/A
	Sepsis, n (%)		0 (0)	0 (0)	0 (0)	N/A
	Death, n (%)		0 (0)	0 (0)	0 (0)	N/A
	Time of hospitalization (days), median (IQR)		22.0 (14.0-28.0)	17.0 (11.0-28.0)	22.0 (15.5-26.5)	.53
	Time from onset to diagnosis (days), median (IQR)		9.5 (6.3-17.0)	4.0 (2.0-17.0)	10.0 (7.5-17.0)	.09
	Time of viral shedding after onset of symptom (days), median (IQR)		25.0 (19.0-29.0)	24.0 (14.0-26.0)	26.0 (20.0-29.0)	.21
	Redetected positive for discharged patients, n/N (%)		2/30 (7)	2/11 (18)	2/19 (11)	.61

^a^N/A: not applicable.

Of the pregnant women, 2 were classified with severe illness on admission, neither progressed to critical illness. In addition, no miscarriage was observed in the pregnant women. Out of 11 pregnant women, 10 underwent cesarean sections (2 had preterm birth; Multimedia Appendix 1).

The median length of hospitalization for the pregnant and nonpregnant groups was 17.0 and 22.0 days, respectively. In addition, the median interval from onset to diagnosis of SARS-CoV-2 were 4.0 and 10.0 days, respectively. The median duration of viral shedding after COVID-19 onset was 24.0 and 26.0 days, respectively. All patients who recovered from COVID-19 were placed in an isolation center for quarantine for a period of 2 weeks. SARS-CoV-2 was redetected in 11 pregnant and 19 nonpregnant women after discharge. SARS-CoV-2 was tested positive in 2 (18%) pregnant women and 2 (11%) nonpregnant women, and all were readmitted at hospitals for COVID-19 treatment (Table 3).

## Discussion

### Principal Results

This study retrospectively analyzes the epidemiological, clinical, laboratory, and imaging characteristics, and clinical outcomes of 43 women of childbearing age infected with COVID-19, including 17 pregnant women and 26 nonpregnant women. As of March 28, 2020, none of the patients involved in this study developed severe pneumonia or died. Based on our findings, there is currently no evidence indicating that pregnant women are more susceptible to the occurrence and severe adverse outcomes of COVID-19 than the general population.

A woman’s body is in a highly immunosuppressive state after pregnancy, and the anatomy, physiology, and biochemistry will always change. For example, the immunity of T lymphocyte changes, the oxygen consumption increases, and the diaphragm elevates, which increases the risk of respiratory infection of pregnant women [9,10]. Studies during the outbreak of influenza virus and SARS-CoV have demonstrated that pregnant women are more susceptible to severe illness. In the outbreak of the “Spanish flu” in 1918, 675,000 people died, with an overall mortality rate of 1%-2%, while 27% of pregnant women died, and the mortality rate of pregnant patients reached 50% or higher when complicated with secondary bacterial pneumonia [18,19]. In the outbreak of SARS in 2003, among 12 pregnant women diagnosed with SARS, 6 (50%) needed to be admitted to the ICU, 6 (50%) underwent mechanical ventilation, and the mortality rate was 25% [11]. Another study reported that 6 of 10 (60%) pregnant women with SARS were admitted to the ICU, 4 (40%) underwent mechanical ventilation, 3 (30%) progressed to renal failure, 2 (20%) progressed to secondary sepsis, 2 (20%) progressed to secondary DIC, and the mortality rate reached 30% [12]. During the COVID-19 outbreak in 2019, one study reported that none of the 9 pregnant patients progressed to critical illness or death [14]. Of the 16 cases of pregnant women with COVID-19, one was classified as severe but did not develop severe adverse outcomes in the later stage [20]. This is consistent with our findings that none of the pregnant women with COVID-19 developed severe adverse outcomes. Although critical pneumonia and death have not been reported in pregnant women, we should still be alert to the possibility of pregnant women developing severe adverse outcomes considering the high similarity of genomic sequence between SARS-CoV and SARS-CoV-2 [6].

### Comparison With Prior Work

In this study, none of the 17 pregnant women had a history of exposure to the Huanan Seafood Market, 53% (n=9) had routine prenatal care within 2 weeks before onset, and 24% (n=4) had a family cluster of COVID-19. Therefore, during the epidemic, it was recommended that pregnant women delay their routine prenatal care for safety, unless it was necessary, or to use an online clinic to reduce the risks of nosocomial infection. Similar to previous studies, common symptoms at the onset of COVID-19 were fever and cough, and less common symptoms were expectoration, chest tightness, and diarrhea [14,15,21,22]. Notably, the onset of symptoms for several pregnant women were atypical, given that they had no fever or cough before diagnosis and only symptoms related to pregnancy were observed, including abnormal pain, vaginal bleeding, and increased or reduced fetal movement, which indicated that attention should be paid to the occurrence of atypical symptoms in pregnant women. Laboratory findings were significantly different in hematological parameters between the two groups. Leukocytosis featured prominently in pregnant patients [14,15], while leukopenia featured prominently in nonpregnant patients [21,22]. Lymphopenia is likely to occur in both groups. Elevated concentration of hs-CRP, D-dimer, and liver enzymes (including ALT, AST, LDH, and ALP) in pregnant patients with COVID-19 were observed; none of them developed liver failure or coagulation disorders. Recently, a study of 274 cases of patients with COVID-19 found that patients who were deceased generally had markedly higher level of CRP and LDH than recovered patients [22]. Therefore, the possibility that pregnant women with COVID-19 develop severe adverse outcomes cannot be eliminated. Additionally, a certain proportion of SARS-CoV-2 and influenza A virus coinfection were shown in the two groups. Given the similar clinical manifestations caused by the two viruses and a relatively low positive rate for the SARS-CoV-2 RNA test, it is recommended that a comprehensive assessment including epidemiological exposure, symptoms, laboratory, and imaging tests are necessary to the diagnosis of COVID-19.

Currently, vaccine or specific treatment for COVID-19 infection is absent. The majority of patients received antivirals such as arbidol and oseltamivir, and empirical antibiotics treatment, while few patients received glucocorticoid and immunoglobulin therapy. Arbidol is an antiviral agent with a unique mechanism of action targeting the S protein/angiotensin-converting enzyme 2 interaction and inhibiting membrane fusion of the viral envelope [23]. In vitro data suggested its activity against SARS [24]. In addition, a nonrandomized study of 67 patients with COVID-19 reported that, compared with arbidol-untreated patients, arbidol-treated patients with a treatment for a median time of 9 days showed a lower mortality rates (0% vs 16%) and higher discharge rates (33% vs 19%) [25]. However, limited data are available on the safety of medications used during pregnancy. Oseltamivir is a neuraminidase inhibitor approved for the treatment of influenza, but it has no documented in vitro activity against SARS-CoV-2. Antibiotics were used routinely after operation to prevent secondary bacterial infections. Routinely systemic corticosteroids for treatment of COVID-19 is not recommended [3]. A large proportion of nonpregnant women used antitussive drugs in this study, which was related to higher proportions of cough (77%) during disease progression (Multimedia Appendix 1). Supportive therapy and oxygen therapy are important for the management of COVID-19 [3].

No significant difference in the length of hospitalization for patients with COVID-19 patients was observed in the two groups. Notably, both pregnant and nonpregnant recovered patients tested positive for SARS-CoV-2 RNA during isolation. Fortunately, none of them experienced symptoms again or developed severe pneumonia. In a case series including 4 patients with COVID-19 who had 3 repeated qRT-PCR after discharge or discontinuation of quarantine, 4 (100%) redetected positive for SARS-CoV-2 RNA. All of them did not have contact with patients with suspected or confirmed COVID-19, and no family member was infected [26]. Thus, at least a proportion of recovered patients may still be virus carriers, and quarantine is still indispensable even after the patient with COVID-19 is discharged.

### Limitations

Our study has some notable limitations. First, this study is limited by its small sample size. More cases of infection from COVID-19 should be used for analysis. Second, only 1 pregnant woman in her first trimester and 3 in their second trimester were included in this study. The effect of COVID-19 on mother and fetus in early pregnancy still needs to be clarified. Third, this is a retrospective study; the uncertainty of the exact dates and related information on exposure (recall bias) might have an inevitable impact on assessment. Fourth, this study only included pregnant women and nonpregnant women; another group of healthy pregnant women should be included to assess pregnant outcomes of mother and fetus, and intrauterine vertical transmission potential of COVID-19.

### Conclusion

In this study, the clinical outcomes of pregnant women with COVID-19 appeared good, and none of the patients developed severe adverse outcomes. Additionally, the epidemiology of pregnant women with COVID-19 was complicated, and nosocomial infection cannot be underestimated. Fever and cough were the most common onset of symptoms in pregnant women. Notably, pregnancy-related symptoms (ie, abdominal pain, vaginal bleeding, increased or decreased fetal movement) might be the specific onset of symptoms for pregnant women with COVID-19. Quarantine is still needed after hospital discharge, as a small proportion of recovered patients may still be virus carriers. In conclusion, early detection and active management effectively helps in the risk of developing severe pneumonia and death in pregnant women with COVID-19.

## Appendix

Multimedia Appendix 1 Supplementary material.

## Abbreviations

ALT: alanine aminotransferase
AST: aspartate aminotransferase
CFR: case fatality rate
COVID-19: coronavirus disease
CT: computed tomography
DIC: disseminated intravascular coagulation
ELISA: enzyme-linked immunosorbent assay
hs-CRP: high-sensitivity C-reactive protein
ICU: intensive care unit
LDH: lactate dehydrogenase
MERS-CoV: Middle East respiratory syndrome–related coronavirus
qRT-PCR: quantitative real-time reverse transcription polymerase chain reaction
SARS-CoV: severe acute respiratory syndrome–related coronavirus
SARS-CoV-2: severe acute respiratory syndrome coronavirus 2
WHO: World Health Organization
