# Supplementary material for: Clinical Characteristics and Outcomes of Childbearing-Age Women With COVID-19 in Wuhan: Retrospective, Single-Center Study
Source: J Med Internet Res. 2020 Aug 24;22(8):e19642. doi: 10.2196/19642 (PMC7446716; doi:10.2196/19642)
Supplement: Multimedia Appendix 1 [file jmir_v22i8e19642_app1.docx]

**Method of SARS-CoV-2 RNA test:**

Throat swab samples of patients were collected. The total RNA of throat swab samples were extracted by the respiratory sample RNA isolation kit (Biogerm, Shanghai, China). Briefly, 40 μL of cell lysates were transferred into a collection tube followed by vortex for 10 seconds. After standing at room temperature for 10 minutes, the collection tube was centrifugated at 1000 rpm/min for 5 minutes. The suspension was used for SARS-CoV-2 test by quantitative real-time reverse transcription polymerase chain reaction (qRT-PCR). Two target genes, including open reading frame 1ab (ORF1ab) and nucleocapsid protein (N), were simultaneously amplified and tested during the real-time qRT-PCR assay. Target 1 (ORF1ab): forward primer CCCTGTGGGTTTTACACTTAA; reverse primer ACGATTGTGCATCAGCTGA; and the probe 5′-VIC-CCGTCTGCGGTATGTGGAAAGGTTATGG-BHQ1-3′. Target 2 (N): forward primer GGGGAACTTCTCCTGCTAGAAT; reverse primer CAGACATTTTGCTCTCAAGCTG; and the probe 5′-FAM- TTGCTGCTGCTTGACAGATTTAMRA-3′. The real-time RT-PCR assay was performed using a 2019-nCoV nucleic acid detection kit according to the manufacturer’s protocol (Shanghai bio-germ Medical Technology Co Ltd). Reaction mixture contains 12 μL of reaction buffer, 4 μL of enzyme solution, 4 μL of Probe primers solution, 3 μL of diethyl pyrocarbonate–treated water, and 2 μL of RNA template. RT-PCR assay was performed under the following conditions: incubation at 50 °C for 15 minutes and 95 °C for 5 minutes, 40 cycles of denaturation at 94 °C for 15 seconds, and extending and collecting fluorescence signal at 55 °C for 45 seconds. A cycle threshold value (Ct-value) less than 37 was defined as a positive test result, and a Ct-value of 40 or more was defined as a negative test (ref: Tao C, BMJ 2020 doi: 10.1136/bmj.m1091).

**Supplementary Table 1 Clinical classification of COVID-19.**

| Mild | The clinical symptoms are mild, and there was no sign of pneumonia on chest imaging. |
| --- | --- |
| Moderate | These patients had fever and respiratory symptoms. Radiologic assessments found signs of pneumonia. |
| Severe illness | Patients meet any of the following criteria:  (1) Shortness of breath, RR≥30 times / min;  (2) Oxygen saturation≤93% at rest;  (3) Alveolar oxygen partial pressure / fraction of inspiration O2 (PaO2 / FiO2) ≤ 300 mmHg (1mmHg = 0.133kPa).  At high altitudes (above 1000 meters), PaO2 / FiO2 should be corrected according to the following formula: PaO2 / FiO2× [Atmospheric Pressure (mmHg) / 760].  Patients whose pulmonary imaging showed significant progression of lesion > 50% within 24-48 hours should be treated as severe type. |
| Critically illness | Patients meet any of the following conditions:  (1) Respiratory failure requiring mechanical ventilation;  (2) Shock;  (3) Patients combined with other organ failure needed ICU monitoring and treatment. |

**Supplementary Table 2 Clinical features of pregnant and non-pregnant patients with COVID-19. Values are numbers (percentages) unless stated otherwise**

|  |  | **Pregnant COVID-19 patients (n=17)** | **Non-pregnant COVID-19 patients (n=26)** | **p value** |
| --- | --- | --- | --- | --- |
| **Symptoms during the course of COVID-19** | | | | |
|  | Fever, n (%) | 14 (82%) | 23 (88%) | 0.91 |
|  | Chills and rigors, n (%) | 2 (12%) | 5 (19%) | 0.82 |
|  | Headache, n (%) | 0 (0%) | 4 (15%) | 0.25 |
|  | Fatigue, n (%) | 7 (41%) | 13 (50%) | 0.57 |
|  | Sore throat, n (%) | 4 (24%) | 1 (4%) | 0.14 |
|  | Cough, n (%) | 8 (47%) | 20 (77%) | 0.04 |
|  | Expectoration, n (%) | 5 (29%) | 12 (46%) | 0.27 |
|  | Chest pain, n (%) | 1 (6%) | 2 (8%) | 0.70 |
|  | Dyspnea, n (%) | 3 (18%) | 11 (42%) | 0.18 |
|  | Myalgia, n (%) | 4 (24%) | 4 (15%) | 0.79 |
|  | Vomiting, n (%) | 3 (18%) | 3 (12%) | 0.91 |
|  | Diarrhea, n (%) | 5 (29%) | 10 (38%) | 0.54 |

**Supplementary Table 3 Maternal and neonatal outcomes of pregnant patients with COVID-19. Values are numbers (percentages) unless stated otherwise**

|  | **Age, years** | **Gestational age at delivery, weeks plus days** | **Method of delivery** | **Birthweight, g** | **Premature delivery** | **Apgar score**  **(1 min, 5 min)** | **Severe neonatal asphyxia** | **Neonatal death** | **Fetal death or stillbirth** |
| --- | --- | --- | --- | --- | --- | --- | --- | --- | --- |
| Patient 1 | 27 | 39 | C-section | 3090 | No | 8, 9 | No | No | No |
| Patient 2 | 34 | 38+4 | C-section | 3250 | No | 8, 9 | No | No | No |
| Patient 3 | 30 | 39+6 | Vaginal Delivery | 3670 | No | 8, 9 | No | No | No |
| Patient 4 | 34 | 40 | C-section | 3250 | No | 8, 9 | No | No | No |
| Patient 5 | 29 | 40+6 | C-section | 3000 | No | 8, 9 | No | No | No |
| Patient 6 | 34 | 38+2 | C-section |  | No | 8, 9 | No | No | No |
| Patient 7 | 31 | 36+5 | C-section | 2650 | Yes | 8, 9 | No | No | No |
| Patient 8 | 33 | 37 | C-section | 3250 | No | 8, 9 | No | No | No |
| Patient 9 | 30 | 38+5 | C-section | 3350 | No | 8, 9 | No | No | No |
| Patient 10 | 35 | 38+1 | C-section | 3230 | No | 7, 9 | No | No | No |
| Patient 11 | 31 | 36+4 | C-section | 3000 | Yes | 8, 9 | No | No | No |
| n (%) | - | - | - | - | 2 (18%) | - | - | - | - |
